# Host–Parasite Interactions Promote Disease Tolerance to Intestinal Helminth Infection

**DOI:** 10.3389/fimmu.2018.02128

**Published:** 2018-09-20

**Authors:** Irah L. King, Yue Li

**Affiliations:** ^1^McGill University Health Centre, Montreal, QC, Canada; ^2^Meakins-Christie Laboratories, Montreal, QC, Canada

**Keywords:** helminth, infection, immunity, intestine, disease tolerance

## Abstract

Parasitic helminths are among the most pervasive pathogens of the animal kingdom. To complete their life cycle, these intestinal worms migrate through host tissues causing significant damage in their wake. As a result, infection can lead to malnutrition, anemia and increased susceptibility to co-infection. Despite repeated deworming treatment, individuals living in endemic regions remain highly susceptible to re-infection by helminths, but rarely succumb to excessive tissue damage. The chronicity of infection and inability to resist numerous species of parasitic helminths that have co-evolved with their hosts over millenia suggests that mammals have developed mechanisms to tolerate this infectious disease. Distinct from resistance where the goal is to destroy and eliminate the pathogen, disease tolerance is an active process whereby immune and structural cells restrict tissue damage to maintain host fitness without directly affecting pathogen burden. Although disease tolerance is evolutionary conserved and has been well-described in plant systems, only recently has this mode of host defense, in its strictest sense, begun to be explored in mammals. In this review, we will examine the inter- and intracellular networks that support disease tolerance during enteric stages of parasitic helminth infection and why this alternative host defense strategy may have evolved to endure the presence of non-replicating pathogens and maintain the essential functions of the intestine.

“*Generalising about the Nematoda is extremely hazardous. The often cryptic diversity is such that it will frustrate the best of intentions*.”

-WC Clark

## Introduction

Parasitic helminths include a diverse group of intestinal worms that are one of the most successful pathogens of the animal kingdom. Current estimates indicate that over 1.5 billion people and many other agricultural and wild mammalian species are infected with at least one species of intestinal helminth (1). The incredible prevalence of these parasites is largely due to their chronicity of infection—many species can live for years in the host intestine—and the inability of the host to prevent reinfection (2). Although helminth infection is associated with important co-morbidities such as anemia, growth-stunting and digestive disease, infection-induced mortality is relatively rare (<1 per 20,000 individuals) compared to other infectious diseases prevalent in the developing world such as Tuberculosis (~1 in 10) and Malaria (~1 in 100) (3). This low mortality rate is surprising given the fact that the host must accommodate a large (ranging from 1 mm to several meters in length, depending on the species), tissue-invading parasite. The physical characteristics of helminths, their general ability to induce a tissue-healing rather than tissue-destructive immune response and, in some cases, their long-lasting relationship to the host collectively indicate that mammals have evolved to tolerate these parasites.

Tolerance to infection, also called disease tolerance, is a defense strategy by which the host activates intra- and inter-cellular networks to limit the damage incurred by the infectious agent or the immune response without affecting pathogen load (4). Although appreciated in plant biology for decades, the concept of disease tolerance has only recently gained traction as an important mammalian host defense strategy against bacterial, viral and parasitic microorganisms that can occur in combination with or independent of resistance and derive from immune as well as non-immune pathways (5). Disease tolerance is also conceptually distinct from immunological tolerance which involves the unresponsiveness to self or foreign antigens (6). Here we provide a rationale for why disease tolerance is an important defense strategy against helminth infection and include recent data that adds complexity yet excitement to this rapidly evolving research field. Given the diversity of parasitic helminth species, life cycles and susceptible hosts—as eloquently stated by WC Clark (7)—we limit our discussion to the intestinal stage of invasion and/or colonization by nematode species that have co-evolved with rodents and humans and, although they cause significant inflammation and tissue damage during the invasive stage of infection, lead to chronic infection. In addition, we will consider how the parasite itself may promote disease tolerance to ensure its survival and continuation of its life cycle. Finally, we will discuss mechanisms of disease tolerance within the intestine that extend beyond tissue repair programs associated with helminth infection and how they may maintain host fitness in the face of these ancient tissue invaders.

## Type 2 immune-mediated damage control

The most common intestinal parasitic nematodes of humans include the roundworm *Ascaris lumbricoides*, the whipworm *Trichuris trichiura* and the hookworm *Necator americanus*. To propagate their species, these enteric worms have developed mechanisms to invade the host via the skin and/or ensure their survival passage through the oral cavity and stomach until they arrive within the intestinal tissue where they produce eggs that are shed via host feces (8). However, the presence of these large, motile foreign bodies within the epithelial and submucosal layers of the gut disrupts the intestinal architecture and requires tissue remodeling to minimize organ damage and maintain host fitness. These adaptations to infection rely, in large part, on the induction of a type 2 immune response (9).

Studies using naturally-occurring rodent parasites such as *Heligmosomoides polygyrus, Nippostrongylus brasiliensis, Trichinella spiralis*, and *Trichuris muris* in a laboratory setting have demonstrated that upon entry into the intestine, epithelial cells (IECs) are critical for initiating a type 2 immune response. IECs release damage-associated molecules such as ATP as well as the cytokines interleukin (IL)-25 and thymic stromal lymphopoietin that, in combination with diverse sources of IL-33, stimulate tissue-resident type 2 innate lymphoid cells (ILC2s) to produce IL-4, IL-5, and IL-13 (10–14). These quintessential type 2 cytokines rapidly recruit eosinophils and alternatively activated macrophages (AAMacs) with tissue-reparative properties to the site of infection that feedback on to the epithelium to fortify the intestinal barrier by stimulating the production of mucus and anti-microbial peptides as well as enhancing the shedding of dead enterocytes (10). Although the mechanisms by which IECs detect helminth infection remain largely undefined, recent studies demonstrated that succinate, derived from the metabolism of dietary fibers by intestinal *protist* spp., is detected by a specialized subset of IL-25 producing chemosensory IECs called tuft cells. Succinate stimulated tuft cell proliferation (and therefore increased amounts of intestinal IL-25) in a succinate receptor (Sucnr1)-dependent manner. Increased IL-25 stimulated the proliferation of IL-13 producing ILC2s that, in turn, induced goblet cell hyperplasia, intestinal remodeling, and enhanced immunity to subsequent *N. brasiliensis* infection (15–17). Importantly, succinate signals were not required for worm expulsion. These results support the exciting possibility that metabolic signals, while not necessarily critical for host resistance, provide an important pathway used by the host to promote tissue repair and disease tolerance to *N. brasiliensis* infection.

In parallel to ILC2 activation, *T. muris* has been shown to stimulate production of thymic stromal lymphopoietin by IECs that condition intestinal dendritic cells (DCs) en route to the draining lymph nodes to polarize CD4+ T cells into Th2 cells that home to the intestine and amplify the ongoing type 2 response (18). DCs have also been shown during *H. polygyrus* infection to initiate the differentiation of T follicular helper cells that migrate to the B cell follicles and drive a humoral immune response skewed toward the generation of IgG1 and IgE antibody-secreting plasma cells (19, 20). This antibody response enhances the effector functions of macrophages, mast cells and basophil populations through Fc-mediated clearance of cellular debris and release of histamines and eicosanoids that maintain or enhance gut contractility and intestinal blood flow (21–23). Helminth-specific immunoglobulins have also been shown to directly bind and limit parasite motility (21, 24), the latter being necessary for parasite survival.

The importance of the type 2 immunity in response to tissue injury is underscored by a seminal study by Loke and Allen demonstrating that incision of the peritoneal cavity of mice was sufficient to induce transient IL-4Rα-dependent AAMac polarization (25). This work has been recently supported and expanded upon in human vascular disease (26), a zebrafish model of tissue regeneration (27) and mouse models of acute skin (28), liver (29), and muscle injuries (30) where IL-4/IL-13 signals promote clearance of cellular debris and tissue healing by structural cells and AAMacs. Collectively, these results suggest that type 2 immunity is part of a conserved tissue repair program co-opted to limit tissue damage and support barrier integrity during helminth infection. For an in-depth examination of type 2 immunity in tissue repair, we refer you to recent reviews (31, 32).

It is important to note, however, that innate responses to the tissue invasive stages of helminth infection may not be exclusively type 2 immune-driven. For example, Klein and colleagues recently demonstrated that following *H. polygyrus* larvae invasion into the duodenal mucosa, production of a quintessential type 1 cytokine, IFNγ, was important for initiating intestinal crypt remodeling and repair of epithelial barrier integrity (33). Additionally, Bradley and colleagues have described substantial variability in response to TLR2 and TLR4 stimulation of blood monocytes isolated from children infected with *A. lumbricoides, T. trichiura* or hookworms (34). Nevertheless, fecal egg counts positively correlated with production of “pro-inflammatory” cytokine such as TNFα and IL-1β (34). Thus, early responses to helminth infection may simultaneously involve components of a type 1 and type 2 immune response that not only limit microbial invasion during a helminth-induced barrier breach but also promote tissue repair/regeneration and limit tissue damage, yet have minimal effect on parasite burden.

## Disease tolerance as a defense strategy against helminths

The germ theory, posited by Girolamo Fracastoro in the Sixteenth century and proven by Louis Pasteur three hundred years later, stated that microorganisms were the cause of communicable diseases. Although this work led to incredible advancements in our understanding of immunity to infection and the development of antibiotics that have saved millions of lives, it underestimated the diverse functions of microbes in relation to their hosts. It is now well-accepted that mammals have evolved to live in symbiosis with hundreds, if not thousands, of diverse species of bacteria, viruses and fungi (35). Epidemiological data from endemic regions of the world suggest that humans have also developed a mutualistic relationship with helminths. Despite the extraordinarily high prevalence of helminth infection world-wide, the low mortality rate indicates that humans have developed effective strategies, including type 2 immunity, to defend themselves against these parasites. For example, infection with *A. lumbricoides* and *T. trichiura* fail to elicit clinical signs of illness during the intestinal stage of infection except in cases of heavy parasite loads where symptoms likely result from physical obstruction rather than inflammation-induced tissue damage (2). Tolerance to infection is also likely at play in wild rodents as Behnke et al found that at least one of the roundworms *T. spiralis, H. polygyrus* and *T. muris* were present in 90% of wild mice (36). Follow up studies found that trickle infection (repeated administration of <40 larvae) of laboratory mice with *H. polygyrus*, the most common helminth of wild mice, led to asymptomatic chronic infection (37).

Additional evidence that tolerance is an important form of defense against helminth infection are epidemiological studies of “dewormed” human populations (2). Although anthelmintics are very effective at eliminating the primary infection, resistance to re-infection has been rarely observed (2). However, these results have not borne out in laboratory studies of mice as protective immunity to re-infection by the same or heterologous helminth infection can be readily achieved (15, 38). Although the reasons for these disparate results are not entirely clear, one explanation may be the much higher infectious dose typically used in the laboratory setting (>200 larvae or eggs) compared to a lower and repeated trickle infection scenario that occurs in nature. In support of this suggestion, a primary high dose challenge with *T. muris* eggs and *H. polygyrus* larvae promotes worm expulsion whereas lower doses lead to stable or chronic infection (37, 39).

The failure to develop protective immunity to helminths, at least in natural settings of infection, are in part due to the life cycle of the parasite. First, although the specific cell types that are damaged during helminth migration through the intestine are not well-characterized, increased apoptosis of intestinal epithelial cells has been reported during the tissue invasion stage of *H. polygyrus* and *T. muris* infection (11, 40), a process that may simultaneously promote chronic infection and amplify the tissue repair program (discussed in more detail below). Second, type 1 cytokine production including IL-1β and IFNγ that occurs during both *H. polygyrus* and low dose *T. muris* infection (presumably occurring as a result of inflammation-induced cell death or induction of toll-like receptor signals by microbial antigens following a breach in the intestinal barrier) promotes chronic helminth infection by limiting early induction of a protective type 2 response via inhibition of ILC2s and/or Th2 cell activation (39, 41). This work calls for further use of established *in vitro* protocols (42) or the development of new models such as organoid cultures or “tissue-on-a-chip” methodology to allow for more detailed studies on the types of cell stress and/or death that helminths impose on stromal cells and leukocytes. Third, most pathogenic microorganisms including bacteria, fungi, viruses and protozoa possess virulence factors that have direct cytotoxic effects to mediate replication and, ultimately, dissemination. By contrast, intestinal helminths (with the exception of some *Strongyloides spp*.) do not replicate within the host to propagate their species. However, they must reside in the intestine long enough to mature to an egg-laying stage to continue their life cycle. Identifying the fundamental processes of cell and tissue stress that structural components of the intestine undergo to promote an environment hospitable for worm growth will provide a foundation for a more in-depth understanding of disease tolerance to helminth infection.

Several lines of evidence indicate that the benefits of tolerating helminth infection may outweigh the costs in terms of host fitness. For example, type 2 cytokine-mediated goblet cell hyperplasia and expansion of *Clostridia* species in mice infected with *T. muris* can protect susceptible hosts against intestinal inflammation and immunopathology driven by pathobiotic species of *Bacteroides* (43). The same study found that deworming of humans living in regions endemic with helminth infection was associated with an increased *Bacteroides/Clostridia* ratio. Furthermore, in a study of tolerance to macroparasites in a wild vole population, *Gata3* expression—encoding for a transcription factor required for Th2 cell differentiation and ILC2 development and maintenance (44)—by splenocytes and circulating cells was positively associated with parasite burden, animal size and lifespan in older male animals (34). Because the study population was infected with multiple species of macroparasites (e.g., mites, worms, etc), these results cannot causally link helminth infection *per se* to the observed effects on host fitness. Nevertheless, they provide evidence in a natural setting for type 2 immunity in disease tolerance and point to the considerable ecological importance of this defense strategy in wild mammals (45). These results are part and parcel to the concept of concomitant immunity in which the prevention of sterile immunity to one parasite prevents subsequent infection by the same or heterologous pathogens, a phenomenon observed for helminthiasis and other parasitic infections (46, 47). It will be important to determine whether helminths regulate concomitant immunity to other micro- or macroorganisms that impact host health.

In addition to the impact of helminths on intestinal health, their effects extend beyond the gut. Examining a population of Soay sheep in northern Scotland, Hayward et al demonstrated that the amount of weight loss in response to *Strongyloides* burden (as determined by fecal egg counts) was negatively associated with lifetime breeding success (48). These studies suggest that helminth infection may promote the selection of “fitness traits” in mammals. Although the mechanism behind these observations are unclear, helminth infection has important effects on systemic metabolism that may have direct or indirect effects on fecundity. For example, infection of mice with *N. brasiliensis* stimulates the recruitment of IL-4 producing eosinophils to adipose tissue that promote insulin sensitivity and tolerance to glucose (49). Similarly, infection with soil-transmitted helminths including *A. lumbricoides, T. trichiura*, and *N. americanus* has been associated with increased insulin sensitivity (50). As type 2 cytokine production by ILC2s has also been shown to sustain adipose tissue macrophages that regulate thermogenesis and beige fat production (51, 52), it is possible that tolerance to helminth infection evolved to complement physiological mechanisms of core body temperature and metabolic stability in times of variable food abundance and changing seasons in migratory animal species.

## Measuring disease tolerance during helminth infection

**Figure 1:**
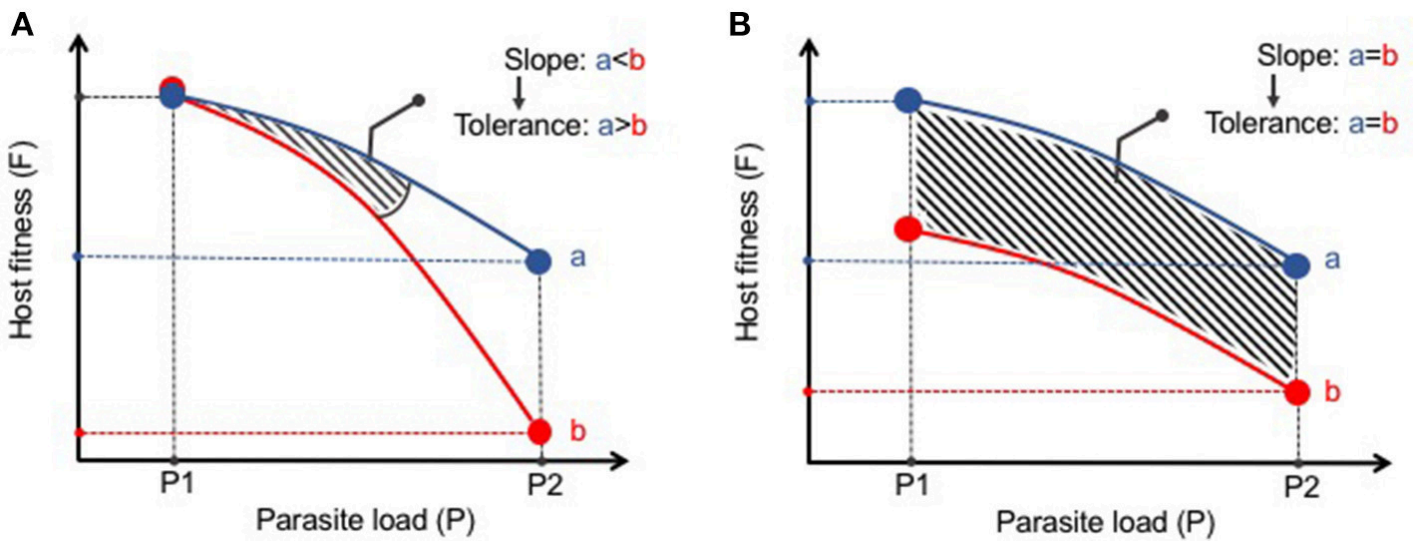
Distinguishing disease tolerance from general vigor during helminth infection. **(A)** Graphical representation of a reaction norm (slope of the curve) where, despite similar starting points (P1), Host B has a greater loss in fitness (i.e., increased morbidity) with increasing parasite load than Host A. Thus, Host A displays greater disease tolerance to infection than Host B. **(B)** Despite differences in host fitness across various parasite loads, the reaction norm between Hosts A and B remains the same. Thus, there is no differences in disease tolerance between A and B, only a difference in general vigor.

The statistical framework for analysis of disease tolerance was initially established in plants in which a “reaction norm” to infection was developed (53). This approach has been more recently supported by Råberg and colleagues as a method to assess disease tolerance in animals (54, 55). A reaction norm in disease tolerance is defined as the health of an animal (or group of animals) across different environments (i.e., pathogen loads) (54). The results can then be plotted with an increasing slope being interpreted as a decrease in tolerance (Figure 1A). This methodology distinguishes tolerance from what Råberg et al refers as “general vigor” or any differences in baseline fitness that may be masked when examining pathogen burden at one point in time (Figure 1B). These strict measures of disease tolerance are difficult to quantify in humans but can be carefully examined in experimental systems in which the response to infection can be assessed over time and infection intensity. As most parasitic worms do not replicate in their definitive hosts, experimental models in which a given number of infectious eggs or larvae are administered results in the same number of adult worms. This relatively stable parasite number provides an excellent opportunity to study changes in tolerance to infection. Nevertheless, it remains important to verify parasite load at different time points or at various infectious doses as persistence of infection can vary depending on the genetic background and environment of the host species (8). A current obstacle in studying host fitness to helminthiasis is that most infections do not elicit such robust clinical phenotypes commonly used as research outcomes such as weight loss, lethargy, or death. In future studies it will be important to expand the breadth and depth of fitness measures (e.g., intestinal and peripheral organ function, serum metabolites, behaviorial abnormalities, etc) in laboratory or natural settings of infection to more effectively define changes to host physiology and better model the co-morbidities associated with human helminth infection.

## Parasite-derived mechanisms that promote tolerance

An important feature of helminths is their potent excretory/secretory (ES) system that not only promotes tissue invasion and acceptance by the host of a large foreign body, but also enhances wound repair, tissue remodeling and evasion or blunting of the inflammatory response (56). These strategies range from inhibition of immune cell signaling (57) to blockade of antigen presenting cell migration (58, 59) to protease secretion that degrade parasite-trapping antibodies (60). Perhaps the most studied mechanism of immunomodulation by helminths is their ability to alter the stimulatory capacity of DCs while enhancing the generation and function of CD4+ T cells with regulatory function (Foxp3^+^ Tregs or IL-10-producing Foxp3^−^ Tr1 cells) (61–63). For example, DCs conditioned with ES products from diverse types of parasitic helminths can promote Th2 responses and limit Th1 responses while also promoting anti-inflammatory IL-10 producing T cells and Tregs (62, 64, 65). Importantly, Treg cells have been shown to promote tissue repair independent of their immunosuppressive abilities via production of amphiregulin, a member of the epidermal growth factor family of cytokines also produced by ILC2s and Th2 cells during helminth infection (66–68). Consistent with these results, several groups have demonstrated that expansion of Treg cells in *T. muris*-infected mice protects from intestinal pathology (69, 70). Moreover, depletion of Foxp3^+^ Treg cells in *H. polygyrus*-infected animals either did not affect or, in some cases, increased adult worm burden and led to increased morbidity and mortality (70, 71). Collectively, these data support a key role for helminth-induced Treg cells in disease tolerance during helminth infection. It has been additionally determined that helminths can promote Treg expansion directly or indirectly through inducing the production of the regulatory cytokine TGFβ (63). TGFβ is a critical component of the wound repair program and promotes the generation of non-stimulatory, tolerogenic DCs (72, 73). Interestingly, *H. polygyrus* secretes a TGFβ mimic that uses canonical SMAD-dependent signaling to promote Treg differentiation while simultaneously enhancing parasite colonization (63). The combined effects of TGFβ signaling on DC function, Treg induction and tissue repair may place TGFβ at a critical nexus of tolerance to helminth infection. Along with TGFβ, another cytokine generally associated with dampening inflammation, IL-10, has been shown to be increased in the context of many helminth infections and limit immunopathology (74, 75). Therefore, helminths have evolved various immune regulatory pathways which have drawn increasing interest for their potential as novel therapies for the treatment of autoimmune and other chronic inflammatory diseases (76).

## Helminth-microbiota crosstalk amplify the immunoregulatory response in the intestine

As intestinal helminths co-habitate with the most abundant and diverse microbial community in the host, important interactions occur between these organisms that reside in the same niche (77). Although commensal bacteria and multicellular helminths occupy very different taxonomic space, they have both responded to evolutionary forces by developing strategies of host immunomodulation. Moreover, it is apparent that these different kingdoms of life have developed a surprising degree of dialogue with a common agenda of establishing a new homeostasis in the host intestinal tract (78). For example, *T. muris* migrates to the proximal colon, the site of greatest bacterial abundance in mammals, where they exploit commensal bacteria for egg hatching and adult worm development (79). In turn, *T. muris* infection alters the gut microbiota and promotes resistance against pathogenic bacteria, an effect dependent on the induction of a type 2 immune response (43). However, initial reports investigating the impact of human *T. trichiura* infection on the composition and function of the gut microbiota have provided mixed results (80, 81). Fricke et al. also reported that a type 2 immune response following *N. brasiliensis* infection in mice reduced abundance of segmented filamentous bacteria (SFB) in the small intestine compared to uninfected controls (82). SFB is a potent inducer of IL-17 production by murine T cells, an immune pathway shown to exacerbate tissue damage at the expense of limiting worm burden (82). In complementary studies, Walk et al. found that *H. polygyrus* infection increased the abundance of *Lactobacillaceae*, a family of lactic-acid producing bacteria with established anti-inflammatory and immune suppressive effects (83). Additionally, helminths could also mediate metabolic changes of the commensal bacteria that promote immunoregulatory functions. Indeed, Zaiss et al. demonstrated that *H. polygyrus* infection enhanced the production of short chain fatty acids (SCFAs) by the intestinal bacteria that have potent ability to amplify Treg cell differentiation (84). In summary, experimental models indicate that helminths and the microbiota influence each other's ability to persist in the mammalian intestinal tract and potentially dampen unwanted inflammatory responses in the intestine. Although studies are emerging that support an impact of helminth on the human gut microbiota, more studies are needed to provide a causal relationship and its impact on tolerance to homologous or heterologous co-infection.

## Intestinal physiology shapes disease tolerance to intestinal helminths

The induction of a type 2 immune response to repair tissue damage can require days to take action. However, intestinal helminths can invade host tissues within the first hours of infection. Thus, the intestine must have intrinsic properties that protect its vital functions prior to a robust immune response. An examination of intestinal physiology may help understand mechanisms by which these organisms parasitize their host niches and inform us about how hosts evolved to tolerate infection.

Phylogenetic studies indicate that parasitic nematodes diverged from their free-living ancestors at least five times during the course of evolution (85). A parasitic lifestyle may have been exploited by helminths during evolution to avoid predators, obtain a consistent source of nutrients and increase fecundity. The conservation of larval developmental stages and the stimuli that promote maturation of diverse nematode species supports this proposition. For example, *in vitro* studies have demonstrated that cholesterol derivatives such as 3-keto bile acid-like steroids (e.g., dafachronic acid) inhibit a state of dormancy (referred to as the dauer stage) at the L3 larval stage in both free-living (*Caenorhabditis elegans*) and parasitic nematode species (e.g., *Strongyloides spp*.) and promote maturation to an adult egg-laying stage (86). Similar bile acid components secreted in the duodenum and re-absorbed in the ileum may provide important cues for larval development *in vivo* (87) while simultaneously possessing immunomodulatory properties. Human intestinal macrophages express the g-protein coupled bile acid receptor TGR5 (i.e. GPR131), expression of which can be enhanced by inflammatory cues such as IFNγ (88). Complementary studies in mice have found that, upon ligand binding, TGR5 activates an AKT-mTOR-dependent pathway that limits toll-like receptor signals and promotes an anti-inflammatory phenotype characterized by increased secretion of IL-10 and decreased production of TNFα (89). Whether a bile acid-macrophage axis contributes to disease tolerance during helminth infection is unknown. In addition, many adult worms feed on host tissue and the rapid turnover of epithelial cells, which is further enhanced during inflammation and infection, provides a rich source of food without directly compromising the integrity of the intestinal barrier. Interestingly, artificially increasing the rate of intestinal epithelial cell death in the absence of overt infection leads to a downregulation of pattern recognition receptors by mononuclear phagocytes and amplifies an anti-inflammatory transcriptional profile of efferocytosing CD64+ gut macrophages including upregulation of TAM family members *Axl* and *Mer* (90). TAM members are not only involved in apoptotic cell sensing but, in the presence of the type 2 cytokines IL-4 or IL-13, enhanced the tissue repair response during pulmonary *N. brasiliensis* infection and experimental colitis (91). Increased IEC apoptosis also increased the ability of CD103+ dendritic cells to induce CD4+ T regulatory cells, a population shown to expand during *H. polygyrus* infection and limit tissue damage without affecting worm burden as mentioned above (91, 92).

Although nutrient availability has been shown to play an important role in tolerance and resistance to bacterial and viral infections, how host or parasite-derived nutrients impacts tolerance to helminthiasis is only beginning to be understood. However, the sharing of (or competition for) metabolites between the host and parasite is not without precedent as iron metabolism by macrophages promotes a “tissue-healing” phenotype during infection whereas blood feeding is an important energy source for the hookworms *N. americanus* and *Ancylostoma duodenale* (93). Whether nutritional immunity contributes to disease tolerance to helminth infection is not well understood. Although competition for nutrients between host cells and the parasite may, in most cases, promote symbiosis, heavy worm burdens may shift the balance toward pathology that lead to co-morbidities associated with helminth infection including malnutrition-induced growth-stunting or cognitive dysfunction. Conversely, an increased consumption of nutrients such as arginine by local infiltrating immune cells that double as worm growth factors, could limit parasite survival. Thus, a metabolic tug-of-war may be a critical mediator of host-parasite co-evolution that has promoted host tolerance to parasitism.

**Figure 2:**
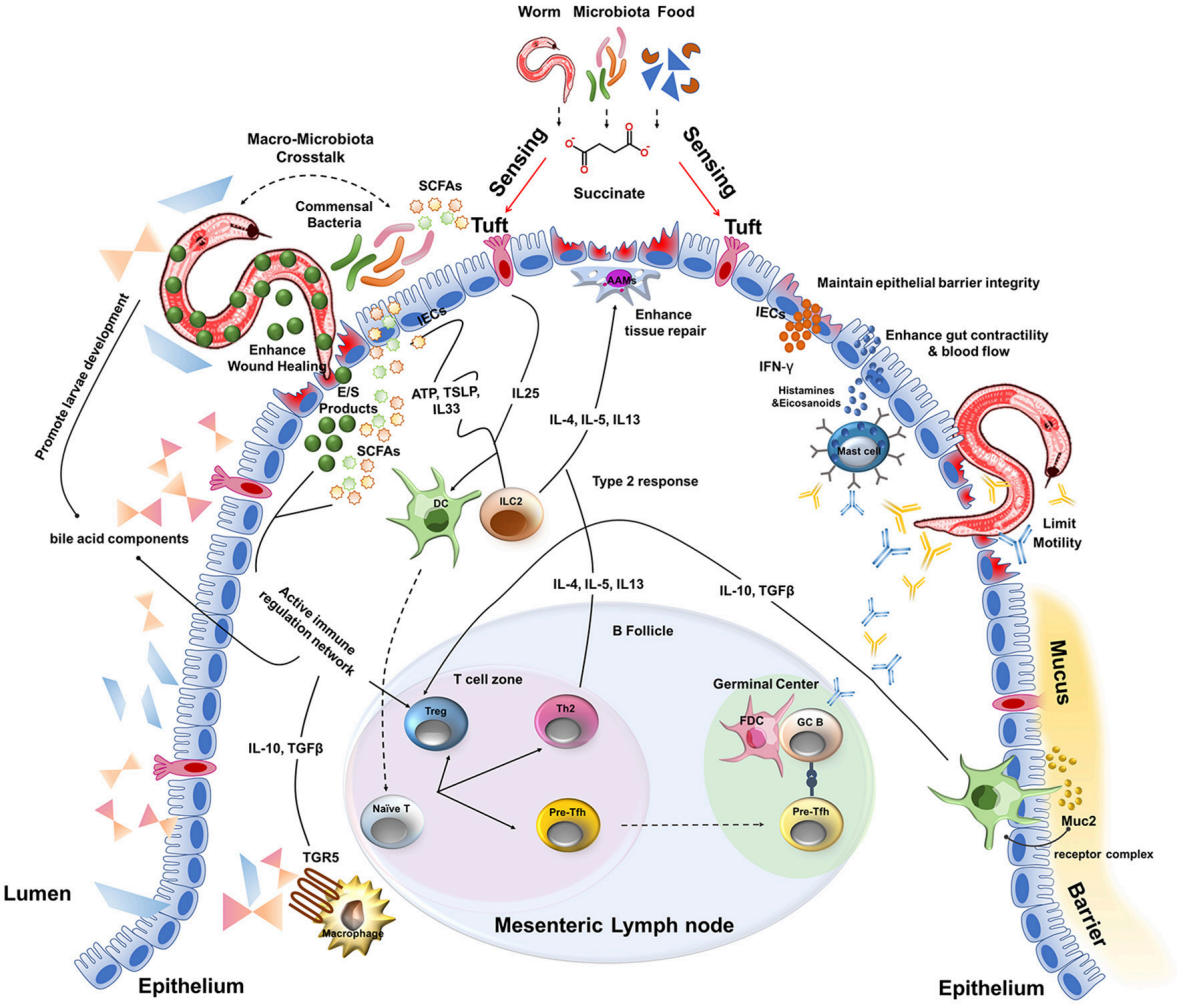
A multitude of host and parasite-derived characteristics impact disease tolerance during the intestinal stages of helminth infection.

Another key component of intestinal physiology is the mucus barrier that lines the length of the intestine. This viscous sheet of glycoproteins covers the epithelium and contains a multitude of viruses (e.g., bacteriophages) and anti-microbial peptides derived from IECs that are toxic to invading bacteria and the commensal microbiota (94, 95). In addition, one of the most abundant mucus proteins is a gel-forming mucin called Muc2 (96). Consistent with the tolerant phenotype of the intestine, resident DCs proximal to the mucus barrier of the small intestine constitutively sample Muc2 through a Galectin-3–Dectin-1–FcγRIIB receptor complex (96). Signaling via this receptor complex inhibits IL-12 production, increases IL-10 and TGFβ production and enhances retinoic acid metabolism by DCs. As a result, Muc2 was able to limit inflammation in a model of experimental colitis as well as promote oral tolerance via induction of Treg cells (96). Although the type 2 cytokine IL-13 produced by ILC2s and Th2 cells is a potent inducer of mucus production by goblet cells and facilitates the “weep and sweep” that can contribute to worm expulsion, the inherent properties of mucus may act as a first line of defense to not only limit bacterial invasion but play an active role in immunomodulation during helminth infection. Collectively, these results suggest that the unique physiology of the intestine complements a type 2 immune response that together provides a highly tolerant ecosystem for host-parasite mutualism and disease tolerance to helminth infection (Figure 2).

## Concluding remarks

Historically, parasitism has been thought to be solely detrimental: the parasite benefits at the expense of host health, with only one “winner” emerging from this interaction. Therefore, developing resistance to these invaders was the conceptual framework that led to great advances in understanding type 2 immunity and its relation to anti-helminth immunity. However, adapting concepts of host defense from studies in plants to a rodent model of malaria infection, Råberg et al. demonstrated that genetic variation in mice can dictate susceptibility to infection without appreciable effects on parasite burden (55). This demonstration of disease tolerance in mammals has now set the stage for investigating the relevance of disease tolerance in other settings of infection. Given that helminth infection almost universally activates type 2 immune pathways yet does not necessarily lead to resistance or protective immunity to re-infection suggests that tolerance is an important, mode of host defense to this unique class of parasitic infection. Although the global morbidity resulting from parasitic helminth infection cannot be understated, increasing evidence suggests that, under certain conditions, helminths may provide a benefit to host fitness. Combining the potential advantages of helminth infection for both host and parasite with the observation that type 2 immunity is a fundamental component of the mammalian response to tissue injury provides a rich example of adaptation between host and parasite that maximizes the survival of both species. Indeed, it is suggested that helminths have interacted with the vertebrate immune system for hundreds of millions of years thus likely shaping the characteristics of both (7, 97). As opposed to other cytokine signaling networks, no individuals have been identified that possess loss-of-function mutations in IL-4Rα or STAT6, the common receptor subunit and downstream transcription factor required for IL-4 and IL-13-induced gene expression. These observations make it tempting to speculate that trait selection is based, in part, on adaptation to helminth infection.

Tolerance to helminth infection also corresponds well with the “hygiene hypothesis” (and the expanded “old friend's hypothesis”) suggesting that diminished exposure to infections or decreased diversity of commensal microorganisms has led to an increased prevalence of allergic (and potentially autoimmune) disease because of defective regulation of the immune system in early life (98). Going forward, a more complete picture of helminth–microbiota interactions and their effects on the host will certainly yield new approaches for the treatment of these “diseases of the developed world”. It will also be important to identify the specific types of tissue damage and cell stress imposed by intestinal helminth infection to understand how the host limits tissue damage and initiates a repair process that is critical for tolerance. Moreover, further investigations will be required to understand the role of intestinal physiology on susceptibility to helminth invasion and its ability to simultaneously minimize immune-driven pathology in the context of tissue damage, a body of knowledge that could be applied to diverse settings of tissue infection and injury. Given the pleiotropic effects that helminths have on the immune system and on host health, tolerance to these parasites may have evolved to provide a unique form of “physiological inflammation” so elegantly conceptualized by Mechnikov over 100 years ago (99).

## Author contributions

YL constructed the figures and wrote the manuscript. ILK conceived of the topic and wrote the manuscript.

### Conflict of interest statement

The authors declare that the research was conducted in the absence of any commercial or financial relationships that could be construed as a potential conflict of interest.
